# Uncoupling of the Femoral Head in a Case of Bipolar Hemiarthroplasty Dislocation

**DOI:** 10.7759/cureus.28716

**Published:** 2022-09-03

**Authors:** Jithin C Davies, Anto A Gopurathingal

**Affiliations:** 1 Orthopedics, Imbichibava Memorial Co-operative Hospital and Research Centre, Malappuram, IND; 2 Orthopedics, Fortis Healthcare, Mumbai, IND

**Keywords:** femur fractures, neck of femur fractures, femoral head, orthopedics, hip, dislocation, uncoupling, bipolar head

## Abstract

Bipolar hemiarthroplasty of the hip is a routinely done procedure in the elderly with neck of femur fractures. An uncommon yet widely recognized complication is the dislocation of hemiarthroplasty. Uncoupling of the femoral head from the stem implant can complicate such dislocations. Such uncoupling requires open reduction. Here, we present a case where the patient sustained a hip dislocation and uncoupling following a bipolar hemiarthroplasty.

## Introduction

Bipolar hemiarthroplasty of the hip is a routinely done procedure in the elderly with neck of femur fractures. Dislocation is a widely reported complication with an incidence rate of 1.2-3.4% [1]. But only a few reports are available in the literature with regard to the uncoupling of the femoral head from its stem.

## Case presentation

**Figure 1:**
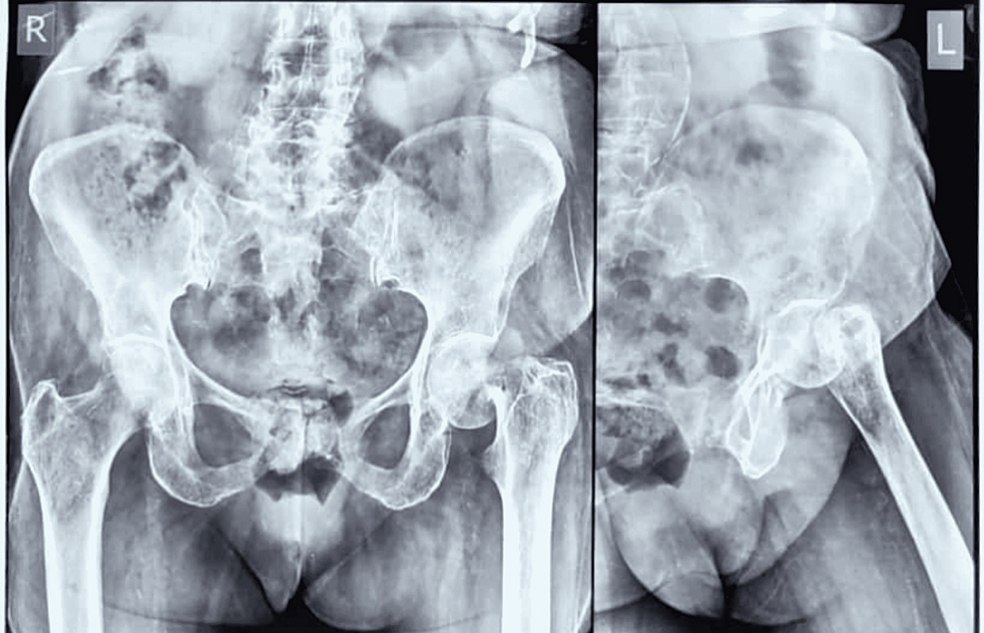
Plain X-ray showing left neck of femur subcapital fracture Pelvis X-ray showing fracture at the base of the neck of femur. Due to the higher age group, hemiarthroplasty was planned.

An 80-year-old lady presented to the emergency department with left hip pain following a trip and fall on a step. A plain X-ray taken at presentation showed a left hip subcapital neck of femur fracture (Figure 1).

**Figure 2:**
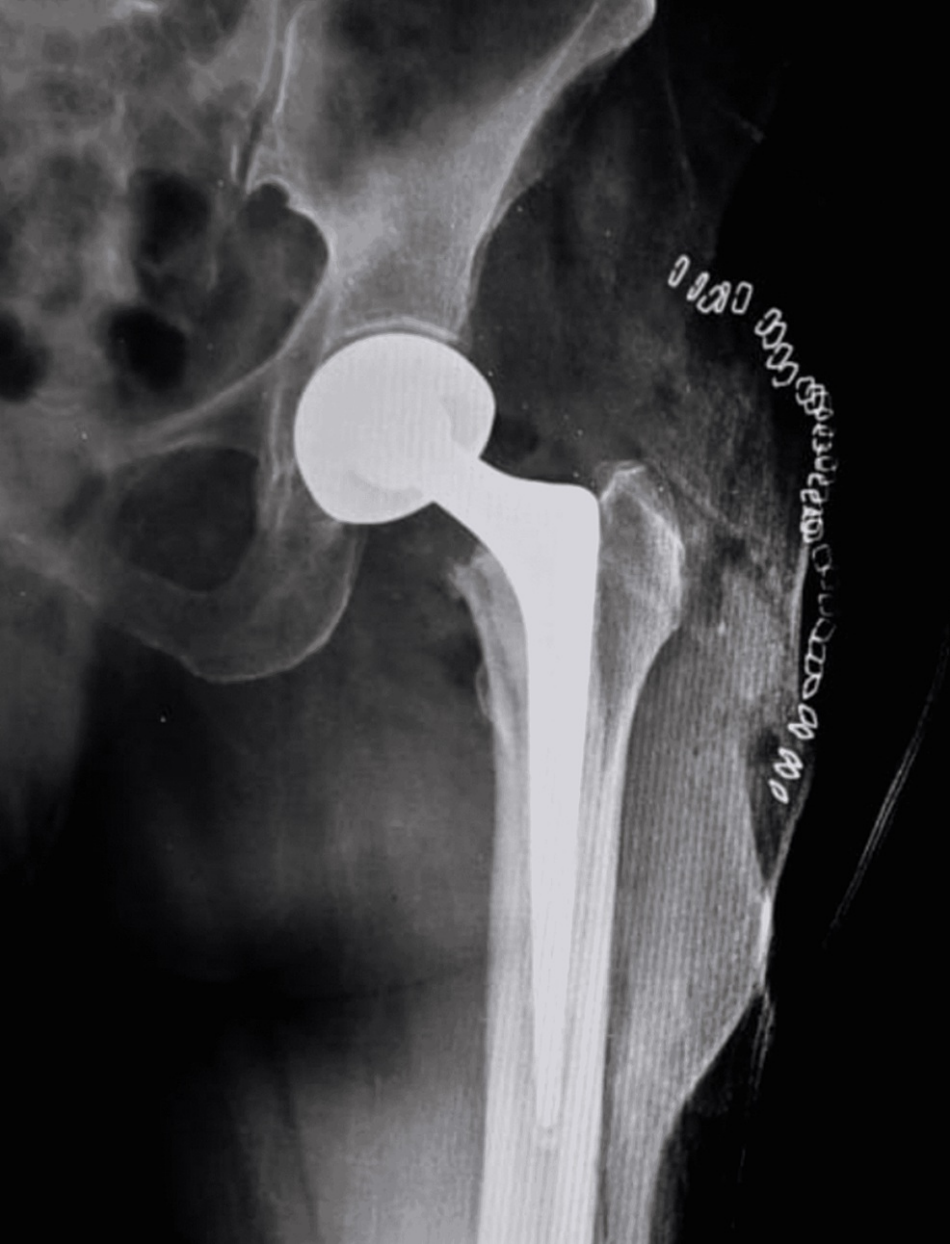
Postoperative X-ray showing bipolar hemiarthroplasty X-ray showing a well-fixed implant. The version appears to be within normal limits.

**Figure 3:**
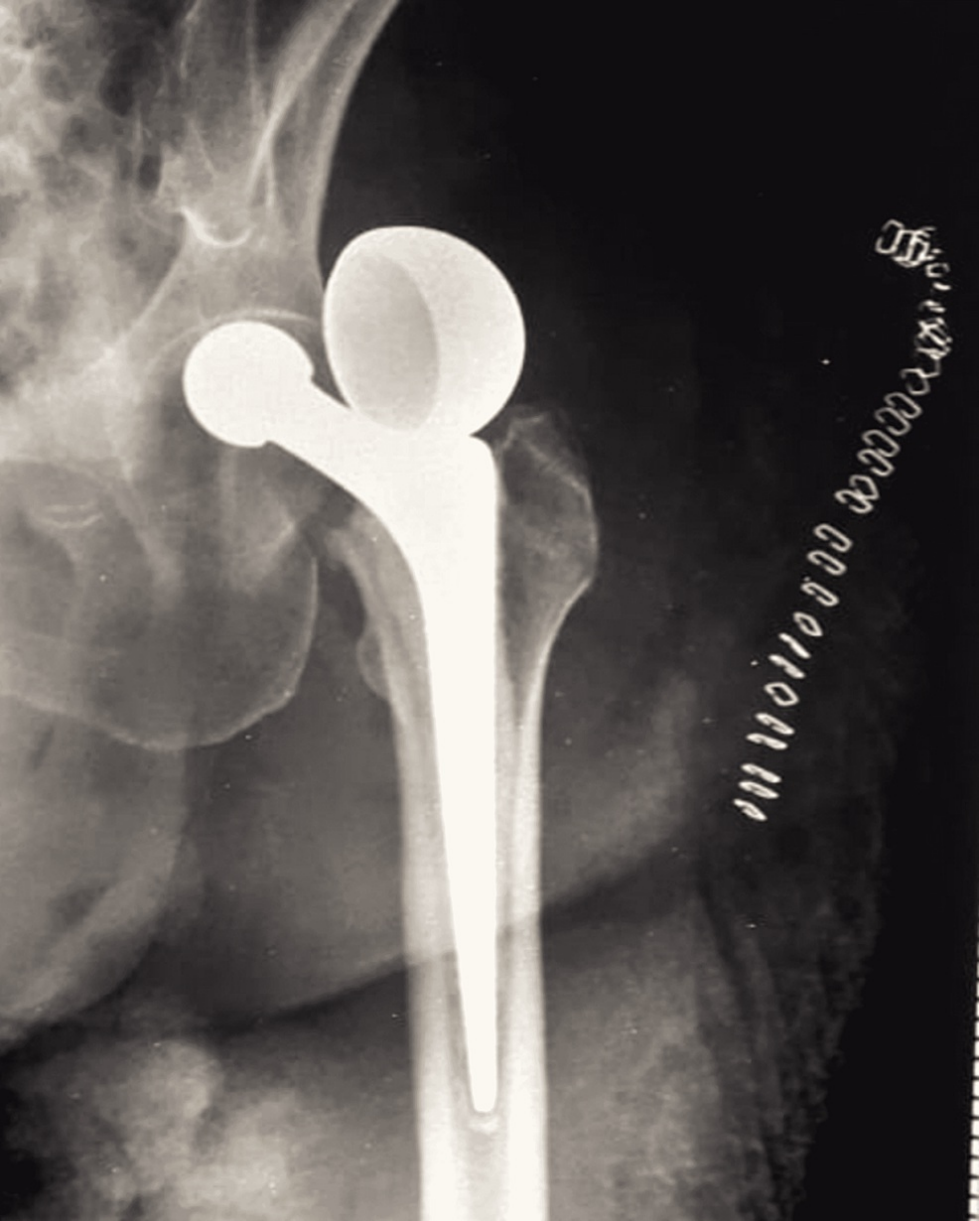
X-ray of the left hip showing dislocation and uncoupling of the femoral head X-ray shows the decoupled bipolar head lying in the supracetabular area.

She underwent a left bipolar hemiarthroplasty (Figure 2). The patient had a history of a cerebrovascular accident five years prior and sustained left-sided hemiplegia. She has been on blood thinners since then. Her postoperative rehabilitation was done with care. While shifting the patient to bed on postoperative day five, the patient complained of severe pain in the left hip. On examination, her left lower limb was externally rotated and shortened. A plain X-ray was done, which showed a dislocation of the head with uncoupling of the head (Figure 3).

**Figure 4:**
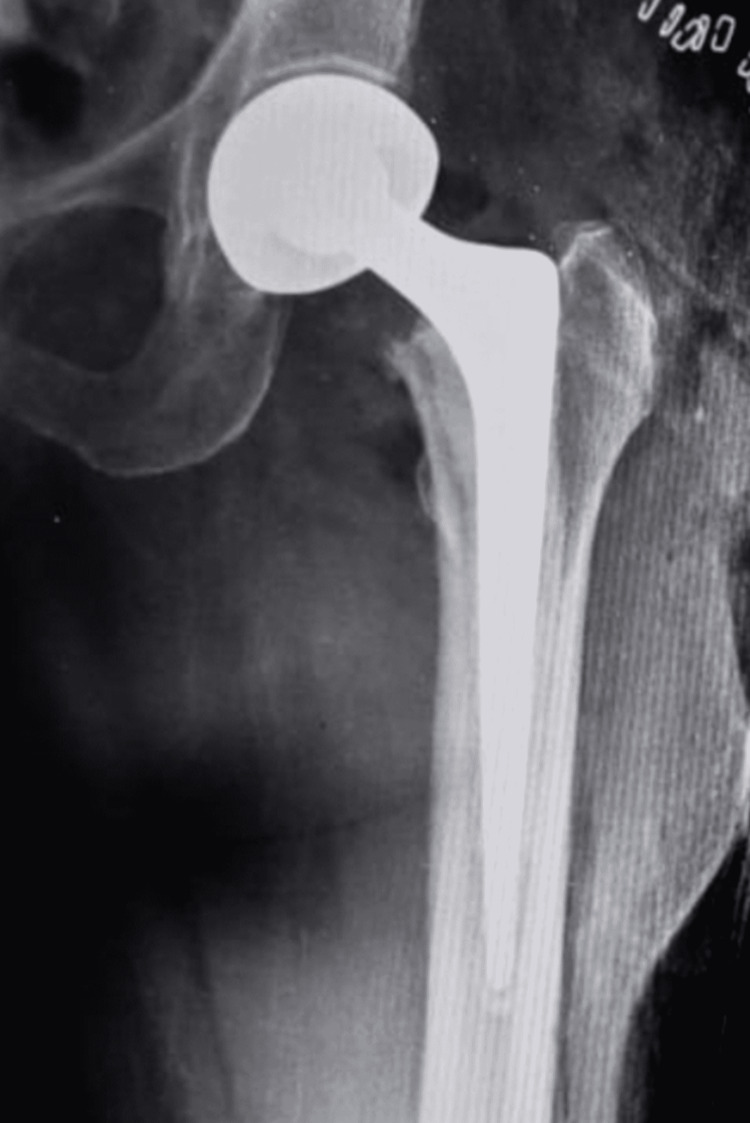
Post-reduction X-ray of the hip X-ray showing reduced head with no other intraoperative events like fractures or loosening.

The patient was immediately shifted to the OR. Closed reduction was not attempted. Open reduction was carried out under spinal anesthesia. The stem appeared well fixed in the proper version; hence, a revision was deemed unnecessary. The hip was reduced and careful repair of the capsule was done. The hip abductor mechanism was repaired meticulously. The postoperative X-ray was satisfactory (Figure 4).

Six months postoperatively, the patient was pain-free and did not have any repeat episodes of dislocation. She was able to ambulate with the help of a walker.

## Discussion

There is no significant difference in the complication rates between unipolar and bipolar hip hemiarthroplasty [2]. Uncoupling of a cup of the bipolar implant is a rare complication with the largest series having only five cases [3]. In the given series, the femoral head remained within the acetabulum, whereas, in our case, the cup had uncoupled and gone out of the acetabulum while the internal femoral head on the stem had located in the acetabulum.

The reason for uncoupling is believed to be the femoral cup component locking to the rim of the acetabulum during dissociation. Weak abductors, inappropriately sized implants, and excessive wear can all cause dissociation [4]. Such uncoupling has also been reported in the attempted reduction of a dislocated head [5].

Such dislocation should be adequately imaged to rule out any other causes or obstructions to reduction. Such dissociations are managed best by open reduction, as seen in our case [6]. Implant malalignment and surgical pitfalls should be reassessed and corrected if found. In our case, we believe the weakened muscle envelope might have given away, causing such a scenario [7]. An intraoperative examination should be done to assess for loosening of the implant or acetabular fractures or labral tears. If there was a further episode of dislocation, then implant revision might have to be carried out.

## Conclusions

Dislocation of bipolar hips in the postoperative period is a rare occurrence in the presence of well-aligned and fixed implantation. Muscle weakness can be an important but overlooked factor that can lead to dislocations in the elderly. Uncoupling of such heads should always be reduced via an open method. Positioning and version of the implant should be checked to assess the need for any revision.
